# Clinical validation of visual LAMP and qLAMP assays for the rapid detection of *Toxoplasma gondii*

**DOI:** 10.3389/fcimb.2022.1024690

**Published:** 2022-09-26

**Authors:** Zhi Cao, Ke Zhang, Dehua Yin, Qiaoya Zhang, Ying Yu, Jianxin Wen, Hongbo Ni

**Affiliations:** College of Veterinary Medicine, Qingdao Agricultural University, Qingdao, China

**Keywords:** *Toxoplasma gondii*, visual LAMP, qLAMP, TaqMan qPCR, rapid detection

## Abstract

Humans are exposed to *Toxoplasma gondii* infection as pet cats gradually become family members and represent an increasing public health risk worldwide. Toxoplasmosis diagnosis constitutes an important measure for disease prevention and control. In this study, real-time fluorescence quantitative loop-mediated isothermal amplification (qLAMP) and visual LAMP detection technologies were established to conduct tests of *T. gondii* based on the membrane DNA extraction method, and the optimal detection mix was determined by adding the protective reagent trehalose and screening the concentrations of Mg^2+^ and dNTPs. Paraffin and lyophilization were used to reduce and even remove aerosol pollution, constructing a detailed anti-contamination protocol. Based on the positive standard plasmid DNA, the LODs of qLAMP and visual LAMP were 92 copies/μL and 92 copies/μL, and the standard curve of qLAMP was Y=2.9503X+20.8992 with R^2 =^ 0.99. The applicability of the qLAMP and visual LAMP assays in disease diagnosis was assessed by evaluating 200 clinical cat faeces samples. The assays showed good diagnostic consistency, with kappa values of 1.0 and 0.99 compared with *Taq*Man qPCR, respectively. Compared with *Taq*Man qPCR, the diagnostic specificity/sensitivity of qLAMP and visual LAMP were 100%/100% and 100%/80%, respectively. The qLAMP and visual LAMP assays reported here are rapid and simple tests without extensive sample preparation and have a short turnaround time within 60 min, making them suitable for point-of-care testing.

## Introduction

Toxoplasmosis, an obligate intracellular parasitic zoonotic disease caused by *Toxoplasma gondii* (*T. gondii*), is widespread around the world and infects almost all warm-blooded animals, including humans (Nasiru Wana et al., 2020b; Matta et al., 2021). It is known to cause reproductive failure, resulting in huge economic losses owing to abortion and weak offspring in food animals (Stelzer et al., 2019; Nayeri et al., 2021). Studies have shown that most animals are infected by the ingestion of food and water with oocysts from cat feces, and humans are infected due to consumption of raw and undercooked meat containing bradyzoites or oocysts (Uddin et al., 2021). Approximately one-third of the human population is exposed to *T. gondii* infection through oral, blood and congenital transmission (Abbas et al., 2020; Nasiru Wana et al., 2020a). Felids, the only final host, are infected by tachyzoites, oocysts, or bradyzoites, while other animals, such as livestock, birds, and fish, can all act as intermediate hosts (Nasiru Wana et al., 2020a; S. Al-Malki, 2021). *T. gondii*-infected cats are involved in the transmission of *T. gondii*, but clinical symptoms of *T. gondii* infection usually go unnoticed and cannot be used for early diagnosis (Boothroyd and Grigg, 2002). Moreover, pet owners are at risk of *T. gondii* infection due to the unknown relationship between animal-to-human transmission and pet feeding. At the same time, stray cats and dogs are adopted as companion pets, and unclear infection status may be responsible for the transmission of *T. gondii* to humans. Therefore, rapid, sensitive, and on-site detection technology of *T. gondii* is vital for early infection warning, which helps prevent animal-to-human transmission and effectively maintain public and veterinary health (Uddin et al., 2021).

The diagnosis of *T. gondii* infection frequently depends on serological testing under laboratory conditions and is limited by shortcomings such as being time-consuming, laborious, a health hazard and requiring skilled personnel (Kotresha and Noordin, 2010; Liu et al., 2015). At present, various molecular biological methods with increased sensitivity and specificity have attracted researchers’ attention and have got a significant achievement in the detection of *T. gondii* (Switaj et al., 2005; Ybanez et al., 2020), such as conventional PCR, quantitative PCR (qPCR), and loop-mediated isothermal amplification (LAMP). PCR has been widely used for *T. gondii* detection since *T. gondii* detection was achieved targeting the B1 gene in 1989 (Burg et al., 1989), and qPCR is capable of identifying low copies of target genes and evaluating disease progression (Teixeira et al., 2013). However, skilled personnel and expensive equipment are indispensable for PCR and qPCR, limiting their application in resource-poor regions and precluding their use at the point of care. LAMP is a relatively novel, less time-consuming and simple diagnostic technology under isothermal conditions (Hegazy et al., 2020), and aerosol contamination and reaction mixture instability are the main barriers for accurate detection. Moreover, the use of chromogenic agents, such as hydroxy naphthol blue (HNB), in the LAMP system allows results determination with the naked eye following a visual colour change from violet to blue, thereby reducing the dependence on equipment (Goto et al., 2009; Sheikh et al., 2020). The sensitivity and specificity of molecular biological methods are also affected by DNA extraction technology (Edvinsson et al., 2004). Extraction of *T. gondii* DNA is time-consuming, tedious and ill-suited under field conditions, also limiting the application of nucleic acid-based molecular biological methods (Liu et al., 2015). In addition, it is not clear that if the DNA in *T. gondii* spores was extracted effectively during the sample treatment, which may lead to faulty detection.

Herein, on the basis of an established membrane-based nucleic acid extraction method, we aimed to develop rapid, sensitive and specific qLAMP and visual LAMP assays for *T. gondii* detection by optimizing the reaction system and determining the procedure, while protective reagents were also used to improve the stability of the LAMP system, achieving point-of-care testing, equipment independence and real-time detection and proposing a novel strategy for *T. gondii* investigation, prevention and control.

## Materials and methods

### Sample and animals

The DNA of *T. gondii*, *Giardia lamblia*, *Trichomonas foetus*, *Cryptosporidium muris* and *Neospora caninum* were stored in Shandong Key Lab of Preventive Veterinary Medicine, College of Veterinary Medicine, Qingdao Agriculture University (QAU). *T. gondii* clinical cat faeces samples were collected from pet hospitals in Qingdao, Shandong province.

### Design of *T. gondii* LAMP primers

The conserved region sequences of *T. gondii* in the SAG1 gene were retrieved from GenBank (GenBank accession no. X14080), and the *T. gondii* LAMP primers were designed by Primer Explorer V5 to amplify a fragment about 202 bp. The oligonucleotide sequences of the primers are shown in **Table 1**. The optimal primers were analysed and screened by Oligo 6.0 and synthesized by Shanghai Sangon Biotech (China).

**Table 1:** *T. gondii*-specific primer sets used in this study.

Pathogen	label	Sequence	Length	Amplification size (bp)
*Toxoplasma gondii* (SAG 1)	F3	TGAGACGCGCCGTCACG	17	202
	B3	GGCTCTGTGAGCGCTGTTTTAG	22	
	FIP	GCAACAAGAGGGGGATCCGGGTGTTTGCCGCGCCCA	36	
	BIP	CAAGTTGTCACCTGCCCAGACACTTGAGAGTGAAGTGGTTCTCCGT	46	
	LF	CCACATCGCAAGAACGACATCAGT	24	
	LB	AGCCGCGGTCATTCTCACAC	20	

### Construction and identification of recombinant plasmid

The SAG1 gene sequences of *T. gondii* were retrieved from GenBank (GenBank accession no. X14080) and synthesized by Shanghai Sangon Biotech, and the recombinant plasmid was constructed by linking it with the pMD18-T vector. The mass concentration of the recombinant plasmid was measured and stored at -20°C after the sequence was identified.

### Preparation of *T. gondii* DNA

Whatman™ FTA™ Elute Cards (GE Healthcare Life Sciences, MA, USA) were used for room temperature collection, preservation, and purification of nucleic acids from samples for detection. Briefly, the faeces samples were treated with 50 μL SEMP (containing 1 M Tris-HCl, 700 mM EDTA, 10% SDS, β-mercaptoethanol and equilibrium phenol) (Solarbio, China) in a 1.5 mL nuclease-free tube (Cao et al., 2020). After full homogenization, 0.2 mg/mL chitinase was added at room temperature for 30 min to remove the impact of spores for DNA extraction. Nucleic acid was absorbed onto FTA elute cards, which were cut into 2 mm discs using a sterile 2 mm Harris punch (WB100039) and a cutting mat, and 2 mm discs were punched out and placed into the homogenate, stirring the nucleic acid adsorption material with tweezers to fully wet it. The FTA discs were washed twice, and nucleic acids were eluted with 20 μL sterile water to obtain *T. gondii* DNA. The concentration of nucleic acids was measured using a NanoDrop2000 and stored at -80°C. Finally, 1 μL of eluant was added to the qLAMP and visual LAMP systems for detection.

### Preembedding and freeze-drying of detection reagents

Tube cap preembedding: Neutral red (Solarbio, China) was added to the tube cap of PCR tubes and dried at room temperature. Then, paraffin at 52°C to 54°C was added to cover with neutral red, finishing the tube cap preembedding process.

Reagents preembedding: Mixed detection reagents, specific primers and protectants were added to the PCR tubes and placed in a freeze dryer. Lyophilization was started with prefreezing for 1 h below −40°C and then vacuumed to 0.08–0.10 mbar, followed by heating at −5°C for 1.5 h (Cao et al., 2020).

### qLAMP

First, the reaction system was determined by optimizing the concentrations of Mg^2+^ (2 mM, 4 mM, 6 mM, 8 mM) (NEB, USA) and dNTPs (1.0 mM, 1.2 mM, 1.4 mM, 1.6 mM) (NEB, USA). The best reaction system of qLAMP was determined by analysing the reproducibility and the Ct value. Each run contained diluted *T. gondii* positive standard plasmid (9.2×10^6^ copies/μL) and a negative control without plasmid. The amplification was carried out in the C1000 thermal cycler (BioRad, United States) with a reaction condition of 65°C for 1 min, totally 60 cycles.Three different rooms were used for template preparation, qLAMP master mix preparation, and amplification by qLAMP to circumvent any carry-over contamination with amplified products. The optimal reaction system and conditions were determined as shown in **Table 2**.

**Table 2:** Details of preembedded reagents for detection.

	The reactions (30 μL)
Detection reagent	15× Bst 2.0 WarmStart^®^ DNA Polymerase, 0.1 μL; 10× Bst buffer, 3 μL; 25 mM dNTPs, 1.68 μL; 100 mM MgSO_4_, 2.4 μL; 100 mM Eva Green, 0.375 μL; D-(+)-Trehalose dihydrate (g/mL), 3 μL
Primer	100 μM FIP, 0.48 μL; 100 μM BIP, 0.48 μL; 100 μM LF, 0.24 μL; 100 μM LB, 0.24 μL; 100 μM F3, 0.06 μL; 100 μM B3, 0.06 μL
Template	*T. gondii* DNA, 1 μL
Freeze-dried protective agents	Gelatine (g/mL), 0.864 μL; BSA (g/mL), 0.576 μL; Thiourea (g/mL), 0.288 μL

Nuclease-free water and tube-cap reagent were not listed in the table, and freeze-dried protective agents were not included in total reaction system.

### Visual LAMP reaction

Different colors were exhibited by the chromogenic agents. The *T. gondii* positive standard plasmid was detected with Neutral Red (Solarbio, China) and concentrations that were the same as those used in the established qLAMP reaction. The reaction mixture was incubated with a reaction condition of 65°C for 1 h. The positive results were indicated by a color change from orange to pink.

### Sensitivity assay

The limit of detection (LOD) was determined for qLAMP and visual LAMP assays. Serial dilutions of *T. gondii* positive standard plasmid were prepared from 9.2×10^7^ copies/μL ~ 9.2×10^0^ copies/μL. 9.2×10^7^ copies/μL ~ 9.2×10^0^ copies/μL series of concentrations were measured for qLAMP and visual LAMP. At least three independent experiments of each concentration were assayed, and ddH_2_O served as the negative control.

### Specificity assay

The specificity for *T. gondii* of qLAMP and visual LAMP assays was evaluated using *T. gondii* positive standard plasmid and other parasites DNA (*Giardia lamblia*, *Trichomonas foetus*, *Cryptosporidium muris* and *Neospora caninum*). Three independent experiments for each sample were tested, and ddH_2_O served as the negative control.

### Reproducibility assay of qLAMP

The *T. gondii* positive standard plasmid was diluted from 9.2×10^6^ to 9.2×10^4^ copies/µL. Each concentration was repeated 3 times, and a one-time qLAMP assay was conducted to perform the intrabatch repeatability test by comparing the standard deviation (SD) and the coefficient of variation (CV). Three qLAMP assays using diluted *T. gondii* positive standard plasmid were conducted to perform the interbatch repeatability test by comparing the standard deviation and the CV.

### Comparison of qLAMP and visual LAMP with *Taq*Man qPCR using clinical samples

The diagnostic applicability of visual LAMP and qLAMP assays was evaluated by testing 200 suspected *T. gondii* clinical cat faeces samples collected from pet hospitals in Qingdao, Shandong province. The cat faeces samples were detected by visual LAMP, qLAMP and *Taq*Man qPCR (Liu et al., 2015). The feasibility of visual LAMP and qPCR was evaluated by measuring the diagnostic specificity (DSp), diagnostic sensitivity (DSe) and degree of agreement compared with *Taq*Man qPCR.

### Statistical analysis

The calculation of the diagnostic sensitivity (DSe) and diagnostic specificity (DSp) between the two methods was based on the following formula. DSe = TP/(TP + FN) and DSp = TN/(TN + FP), where TP means true-positive cases, FN means false-negative cases, TN means true-negative cases, and FP means false-positive cases. Kappa = (Po – Pc)/(1 – Pc), where Po is the proportion of observed agreements and Pc is the proportion of agreements expected by chance. The degree of agreement was quantified by Cohen’s kappa coefficient (κ), with the following definitions: no agreement (κ ≤ 0), poor agreement (κ = 0.01 – 0.20), fair agreement (κ = 0.21 – 0.40), moderate agreement (κ = 0.41 – 0.60), substantial agreement (κ = 0.61 – 0.80) and perfect agreement (κ = 0.81 – 1.00) (Sim and Wright, 2005). Precision was evaluated by obtaining mean time-to-detection values and standard deviations (SD) of each set of replicates at a given concentration and calculating coefficients of variation (CV = SD/Mean).

## Results

### Identification of the qLAMP reaction

**Figure 1:**
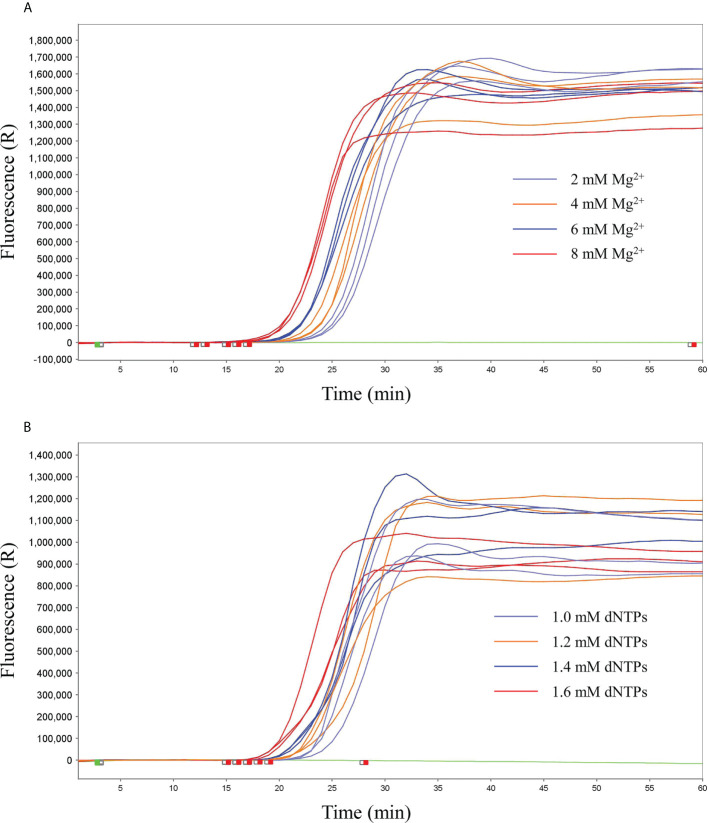
Screening of optimal concentrations of Mg^2+^ and dNTPs. **(A)** Screening of optimal concentrations of Mg^2+^, 2 mM, 4 mM, 6 mM, and 8 mM. **(B)** Screening of the optimal concentrations of dNTPs, 1.0 mM, 1.2 mM, 1.4 mM, and 1.6 mM.

Preliminary experiments were performed only with primers in the absence of any templates to evaluate the negative control precision and revealed no significant primer dimer or self-amplifying reaction. According to the reproducibility and Ct value, the better reproducibility was found with the 8 mM Mg^2+^ (Ct value, 18 ± 0.25) and 1.4 mM dNTPs (Ct value, 19 ± 0.24) in reaction mixture. Therefore, 8 mM Mg^2+^ and 1.4 mM dNTPs were determined as the optimal qLAMP reaction concentrations (**Figure 1**).

### Sensitivity analysis

**Figure 2:**
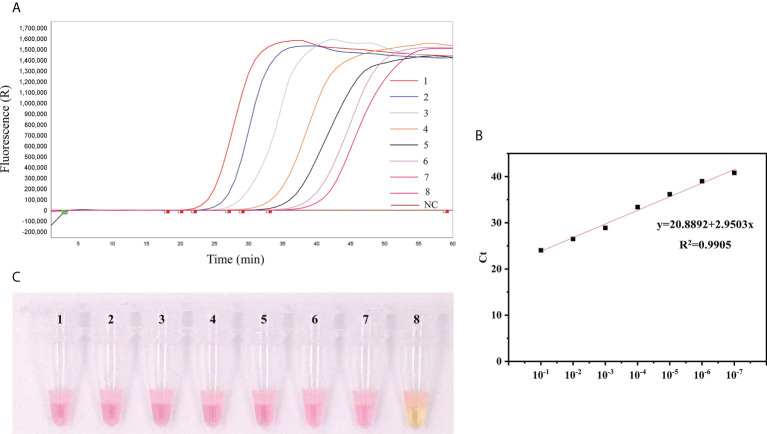
Comparison of the sensitivity of qLAMP and visual LAMP. **(A)** Results of qLAMP analysis. Lanes 1-8: Reaction results from a 10-fold serial dilution of *T. gondii* positive standard plasmids from 9.2 × 10^7^ to 9.2 × 10^0^ copies/µL per reaction; Lane 9: NC (negative control, nuclease-free water), **(B)** Standard curve of qLAMP, **(C)** Results of visual LAMP analysis. Lanes 1-8: 9.2 × 10^7^ to 9.2 × 10^0^ copies/µL.

The sensitivities of qLAMP and visual LAMP (9.2 × 10^7^ ~ 9.2 × 10^0^ copies/µL) assays were assessed using serial dilutions of *T. gondii* positive standard plasmid DNA. All assays efficiently detected lower copies of *T. gondii* DNA (**Figure 2**), and the LODs of the two assays were 92 copies/µL and 92 copies/µL, respectively.

### Specificity analysis

**Figure 3:**
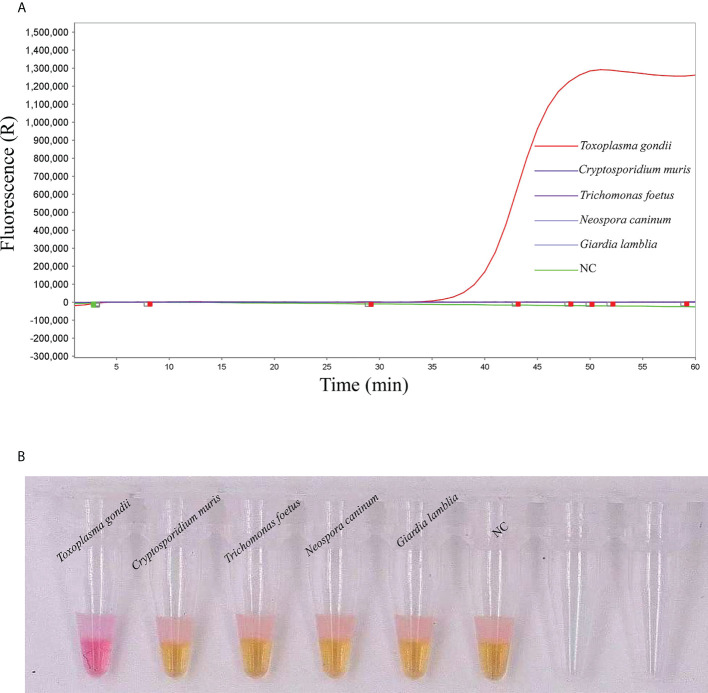
Detection specificity of *T. gondii* positive standard plasmids analysed using qLAMP and visual LAMP. **(A)** Specificity results for qLAMP, **(B)** Specificity results for visual LAMP in detecting *Giardia lamblia*, *Trichomonas foetus*, *Cryptosporidium muris* and *Neospora caninum*. NC, Negative control, nuclease-free water.

We tested the specificity for *T. gondii* positive standard plasmid DNA of qLAMP and visual LAMP assays. *T. gondii-*positive standard plasmid DNA, along with *Giardia lamblia*, *Trichomonas foetus*, *Cryptosporidium muris* and *Neospora caninum*, was used in the specificity test (**Figure 3**). All three assays only amplified *T. gondii*, with no cross-reaction with any of the other parasites.

### Reproducibility analysis

The intrabatch repeatability test and the interbatch repeatability test were measured using the *T. gondii* positive standard plasmid with concentrations of 9.2×10^6^ ~ 9.2×10^4^ copies/μL by qLAMP. The results showed that the reproducibility performance was excellent, and the intrabatch coefficient of variation (CV, 1.34% ~ 1.68%) and interbatch coefficient of variation (CV, 1.25% ~ 1.72%) were less than 2% (**Table 3**).

**Table 3:** Reproducibility analysis for *T. gondii* positive standard plasmid DNA by qLAMP.

Plasmid standard	Plasmid concentration(copies/μL)	Duplicates	Intrabatch reproducibility test		Interbatch reproducibility test	
			$\bar{X}$ ± s	coefficient of variation (CV, %)	$\bar{X}$ ± s	coefficient of variation (CV, %)
*T. gondii*	9.2×10^6^	3	26.51 ± 0.24	0.91	26.57 ± 0.41	1.54
	9.2×10^5^	3	28.79 ± 0.29	1.00	28.78 ± 0.45	1.56
	9.2×10^4^	3	33.50 ± 0.41	1.22	33.12 ± 0.24	0.72

### Performance of qLAMP and visual LAMP for clinical samples compared with *Taq*Man qPCR testing

To evaluate the practical application of qLAMP and visual LAMP to detect *T. gondii*, 200 cat faeces samples suspected to be positive for *T. gondii* were assessed, and the results were compared with those obtained using *Taq*Man qPCR (**Table 4**).

**Table 4:** Comparison of the tests for *T. gondii* positive standard plasmid DNA by qLAMP, visual LAMP and *Taq*Man qPCR.

Assays	Results	*Taq*Man qPCR			Performance Characteristics (%)		Kappa Value
		P	N	Total	DSe	DSp	
qLAMP	Positive	5	0	5	100%	100%	1
	Negative	0	195	195			
Visual LAMP	Positive	4	1	5	80%	100%	0.99
	Negative	0	195	195			

κindex: no agreement (κ ≤ 0), poor agreement (κ = 0.01 – 0.20), fair agreement (κ = 0.21 – 0.40), moderate agreement (κ = 0.41 – 0.60), substantial agreement (κ = 0.61 – 0.80) and perfect agreement (κ = 0.81 – 1.00).

Overall, using *Taq*Man qPCR, 5 samples were confirmed to be positive for *T. gondii* DNA, and 195 samples were confirmed to be negative, with undetermined Ct values. qLAMP detected 5 samples as *T. gondii*-DNA-positive and 195 as negative. Visual LAMP indicated 4 positive and 196 negative samples. Agreement analysis according to clinical sample detection demonstrated that the kappa values between qLAMP and visual LAMP with *Taq*Man qPCR were 1.0 and 0.99, respectively (**Table 4**). Additionally, in comparison to *Taq*Man qPCR, the sensitivity of qLAMP and visual LAMP to identify *T. gondii* was 100% and 80%, respectively, while the specificity of qLAMP and visual LAMP to identify *T. gondii* was 100% and 100%, respectively. Thus, qLAMP and visual LAMP showed perfect diagnostic agreement with *Taq*Man qPCR to detect *T. gondii* in clinical samples.

## Discussion

*Toxoplasma gondii* is considered as one of the most successful eukaryotic pathogens that not only causes huge economic losses to animal husbandry but also harms human health (Liu et al., 2012; Liu et al., 2015). At present, numerous molecular techniques, such as LAMP and qPCR, have been applied for the detection of *T. gondii*, achieving satisfying performance in sensitivity and specificity. However, pet cats are gradually regarded as popular domestic pet in young population, and the owners are willing to spend money and time on cats. Therefore, we aimed to develop a rapid, accurate and at-home detection technology for *T. gondii*, meeting the demand of pet owners.

**Scheme 1:**
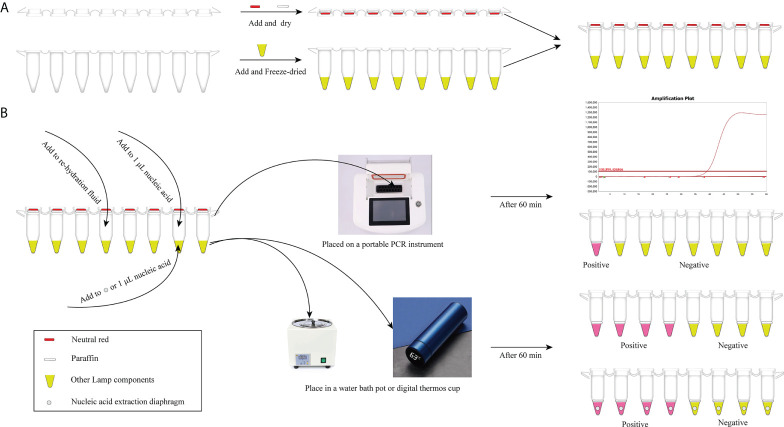
The *T. gondii* detection procedure with qLAMP and visual LAMP. **(A)** Preembedding of neutral red and paraffin and freeze-drying of detection reagents. **(B)** Detection procedure of qLAMP and visual LAMP. qLAMP detection of *T. gondii* is performed in field conditions with a portable PCR instrument and takes within 60 min. Visual LAMP detection of *T. gondii* is performed in field conditions with a heating block or vacuum cup within 60 min.

In our study, considering that the aerosol pollution is troublesome for qLAMP and visual LAMP detection, detailed anti-pollution protocols is one of the highlights. We took full advantage of the PCR tubes and lyophilization, weakening dependency on other packing container (**Scheme 1**). To remove aerosol pollution and improve the accuracy of the detection results, three detailed protocols are designed. Conserved regions (SAG1) were chosen as the target genes for primer design by sequence blasting and sensitivity analysis, improving the specificity of amplification. Then, PCR tube caps and paraffin were utilized to conduct detection reagent preembedding, reducing the nonspecific amplification and preventing aerosol pollution without opening the tube caps. Visual LAMP detection can be completed only with a simple heat block and minimal ease of operation, and qLAMP can be finished with only portable PCR equipment, significantly reducing the time required and achieving a point-of-care test (**Scheme 1**). Above all, a detailed anti-contamination protocol is used for *T. gondii* detection, which is convenient for non-professionals to obtain *T. gondii* detection with no limitations by time and place. Especially, with the gradual increase of dependency for companion pets, convenient, easy-operation and at-home test can attract more attention of pet owners and achieve rapid detection within 60 minutes.

In addition, commercial kits were used to perform DNA extraction, which is time-consuming, tedious, and ill-suited at the POC (Raele et al., 2015). So the membrane technology of nucleic acid extraction is the lightspot to reduce time cost of *T. gondii* DNA extraction, achieving the goal of professional independence and no requirement for other laboratory precision equipment. Given the impact of *T. gondii* spores for DNA extraction, chitinase was used to predigested spores to improve the DNA extraction efficiency.

LAMP assay is more widely used, and reagents are easier to obtain, thereby reducing the diagnosis cost. Moreover, modest equipment and ease of operation are needed, making it convenient for use in field conditions (Mori and Notomi, 2009). However, the stability of the LAMP reaction system is an urgent issue, as colour changes of detection reagents can be performed automatically with temperature changing when chromogenic agents exist, resulting in false-positives and limiting the application of LAMP. Studies have shown that trehalose can conduct nonspecific protective actions for biomolecules by replacing hydrones and weakening molecular movement (Sampedro et al., 2001). In our study, 10% trehalose was used in detection reactions, exhibiting satisfactory reproducibility. Therefore, the protective reagent trehalose was added to the detection reagents, and prelyophilization was conducted to improve the stability of the detection reagents, lengthening the storage time of the detection reagents and achieving long-distance transport. Additionally, the use of trehalose can protect the detection reagents from reacting automatically, circumventing false-positive results.

Different target genes, including SAG1, SAG2, B1 and 529-bp REP, can be identified by LAMP, in which SAG1, SAG2 and B1 target genes are used for early diagnosis and to identify *T. gondii* in human blood samples (Lau et al., 2010; Hu et al., 2012; Qu et al., 2013). The 529-bp REP gene is suitable for reverse transcription LAMP (RT-LAMP) detection in mice and meat samples (Qu et al., 2013). In our previous study, three groups of primers were designed for qLAMP detection targeting SAG1, SAG2 and 529-bp REP genes. We found that lower copies of *T. gondii* can be identified by SAG1 primers, and considering that early diagnosis of in the blood of *T. gondii* infected pigs based on SAG1 LAMP (Wang et al., 2013), therefore, the SAG1 gene was ultimately chosen as the target gene to use in qLAMP for the detection of *T. gondii*.

Herein, qLAMP and visual LAMP assays were developed to quickly diagnose *T. gondii* in clinical cat faeces samples. Our data showed that the specificities of qLAMP and visual LAMP were all 100% (**Figure 3**), and their sensitivities were 92 copies/µL and 92 copies/µL, respectively (**Figure 2**), indicating that these assays based on nucleic acid extraction membrane techniques had good applicability in clinical practice, similar to that of *Taq*Man qPCR. In addition, our data showed that based on the detection sensitivity of a *T. gondii*-positive plasmid, the qLAMP assay could detect 92 copies/µL served as suitable tests of clinical samples with low titres (Liu et al., 2015). Moreover, our validation results showed that these two methods were in perfect agreement with those of *Taq*Man qPCR (with kappa values of 1 and 0.99, respectively) (**Table 4**). In particular, visual LAMP detection can be completed within 60 minutes, by which the speed and simplicity of the methods make visual LAMP ideally suited for molecular applications both within and outside the laboratory, including limited-resource settings (**Scheme 1**).

In conclusion, this was the first application of no-equipment DNA extraction for LAMP technology for *T. gondii* detection. Additionally, trehalose was used to improve the stability of the LAMP reaction system, paraffin was added at preembedding reagents to reduce aerosol pollution, and neutral red was used to achieve visual and point-of-care test detection, minimizing the time cost and providing convenience for pet owners. Our present qLAMP and visual LAMP methods not only have a short turnaround time but also avoid cross-contamination problems and dependence on expensive equipment, which are desirable characteristics amenable to POCT in resource-limited and domestic settings.

## Data availability statement

The original contributions presented in the study are included in the article/supplementary material. Further inquiries can be directed to the corresponding author.

## Author Contributions

Data curation: ZC, KZ, DY and QZ. Formal analysis: JW and YY. Methodology: ZC, KZ, DY, QZ and YY. Project administration: ZC and JW. Supervision: ZC and HN. Writing–original draft: ZC, KZ and DY. Writing–review & editing: ZC and HN. All authors contributed to the article and approved the submitted version.

## Funding

The study was supported by the Shandong Modern Agricultural Technology & Industry System (SDAIT-21-13) and the Research Foundation for Distinguished Scholars of Qingdao Agricultural University (663-1120018 and 665-1120046).

## Conflict of interest

The authors declare that the research was conducted in the absence of any commercial or financial relationships that could be construed as a potential conflict of interest.

## Publisher’s note

All claims expressed in this article are solely those of the authors and do not necessarily represent those of their affiliated organizations, or those of the publisher, the editors and the reviewers. Any product that may be evaluated in this article, or claim that may be made by its manufacturer, is not guaranteed or endorsed by the publisher.
